# The yeast *Wickerhamomyces anomalus* acts as a predator of the olive anthracnose-causing fungi, *Colletotrichum nymphaeae*, *C. godetiae*, and *C. gloeosporioides*

**DOI:** 10.3389/ffunb.2024.1463860

**Published:** 2024-09-17

**Authors:** Mariana Amorim-Rodrigues, Rogélio Lopes Brandão, Fernanda Cássio, Cândida Lucas

**Affiliations:** ^1^ Molecular and Environmental Biology Centre (CBMA), University of Minho, Braga, Portugal; ^2^ Aquatic Research Network (ARNET), CBMA, University of Minho, Braga, Portugal; ^3^ Cellular and Molecular Biology Laboratory, Federal University of Ouro Preto, Ouro Preto, MG, Brazil; ^4^ Institute for Science and Innovation on Bio-Sustainability (IB-S), University of Minho, Braga, Portugal

**Keywords:** BCA (biocontrol agent), antagonistic yeast, *Wickerhamomyces anomalus* LBCM1105, olive anthracnose, *Colletotrichum nymphaeae*, *Colletotrichum godetiae*, *Colletotrichum acutatum* complex, *Colletotrichum gloeosporioides*

## Abstract

Olive tree anthracnose is caused by infection with *Colletotrichum* fungi, which in Portugal are mostly *C. nymphaeae*, *C. godetiae*, and *C. gloeosporioides* s.s. Severe economic losses are caused by this disease that would benefit from a greener and more efficient alternative to the present agrochemical methods. Yeasts are serious candidates for pre-harvest/in field biocontrol of fungal infections. This work identified the yeast *Wickerhamomyces anomalus* as a strong antagonizer of the three fungi and studied *in vitro* this ability and its associated mechanisms. Antagonism was shown to not depend on the secretion of volatile compounds (VOCs), or siderophores or any other agar-diffusible compound, including hydrolytic enzymes. Rather, it occurred mostly in a cell-to-cell contact dependent manner. This was devised through detailed microscopic assessment of yeast-fungus cocultures. This showed that *W. anomalus* antagonism of the three *Colletotrichum* proceeded through (i) the adhesion of yeast cells to the phytopathogen hyphae, (ii) the secretion of a viscous extracellular matrix, and (iii) the emptying of the hyphae. Yeasts ultimately putatively feed on hyphal contents, which is supported by light microscopy observation of MB and PI co-culture-stained samples. Accordingly, numerous *W. anomalus* cells were observed packing inside *C. godetiae* emptied hyphae. This behaviour can be considered microbial predation and classified as necrotrophic mycoparasitism, more explicitly in the case of *C. godetiae*. The results support the prospect of future application of *W. anomalus* as a living biofungicide/BCA in the preharvest control of olive anthracnose.

## 1 Introduction

Olive tree (*Olea europaea* subsp. *europaea*) derived products, olives, and olive oil are of major economic relevance mostly to the Mediterranean region, though it is spreading to other regions, meeting an increasing market demand (Zion Market Search, 2024). Olive trees are susceptible to several pests and diseases, the more severe being infestation with the olive fruit fly *Bactrocera oleae* (Marchini et al., 2017), leprosy caused by the bacteria *Xylella fastidiosa* (Sicard et al., 2018), and anthracnose caused by a consortium of fungi from the *Colletotrichum* genus (Talhinhas et al., 2009; 2009; 2018; Cabral et al., 2024). The latter, while not compromising the survival of the tree, severely affects crop production, pre- and post-harvest, and oil quality (Cacciola et al., 2012; Talhinhas et al., 2005; Peres et al., 2021; Romero et al., 2022).

Virulence and prevalence of olive anthracnose (OA) can be influenced by fungal strain, olive tree cultivar, and environmental factors (Peres et al., 2005; Moral et al., 2009; Talhinhas et al., 2011; Garcia-Lopez et al., 2023). *Colletotrichum* are hemibiotrophic fungi, presenting a biotrophic and a necrotrophic phase. Both the novel and resurging infections of the trees occur during the flowering season (Peres et al., 2005; Moral et al., 2009; Cacciola et al., 2012; Moreira et al., 2023), coinciding with the biotrophic stage of the fungi which occurs through spring and summer. The fungus develops asymptomatically inside the infected flowers and developing fruits. In autumn, the mild and humid weather, coupled with the ripening of the fruits, triggers the fungus’s necrotrophic stage and the consequent development of OA symptoms (Moral et al., 2008; Cacciola et al., 2012; Garcia-Lopez et al., 2023). At this point, if the weather conditions are conducive and the inoculum pressure is high, the conidia can germinate and cause secondary infections, greatly increasing yield losses (Moral et al., 2008; Cacciola et al., 2012). OA leads to premature drop of the drupes or their mummification, remaining in the tree as an inoculum reservoir for resurging infections in the next flowering cycle (Peres et al., 2005; Moral and Trapero, 2012; Sergeeva, 2014; Talhinhas et al., 2018).

Multiple methods of disease management are applied in the control of OA, including early harvesting and use of late-ripening cultivars, severe pruning of the trees, changes in orchard design, and replacement of existent trees with resistant cultivars (Rosa-Magri et al., 2010; Cacciola et al., 2012; Romero et al., 2022). However, the most common strategy is the application of chemical fungicides, namely copper-based fungicides (Materatski et al., 2018) or other drugs such as dithiocarbamate (Ziram), azoles, and strobilurins (Cacciola et al., 2012; Moral et al., 2018; Romero et al., 2022), the efficacy of which varies considerably according to plant cultivar, disease severity, climate, and application timing and frequency (Cacciola et al., 2012; Garcia-Lopez et al., 2023). Importantly, repeated utilization of chemical fungicides tends to induce fungal resistance while causing environmental contamination of soils and underground water through irrigation or rain runoffs. Increasingly, public concern over pesticide-promoted environmental contamination and health problems is pushing for decreased utilization of agrochemicals. This has generated a new market demand for more sustainable and effective alternative ways to control phytopathogens, which include the use of natural microbial antagonists, biocontrol agents (BCAs), or their by-products (Sellitto et al., 2021; Oztekin et al., 2023).

The fungal species responsible for OA in the Northern Hemisphere are *C. gloeosporioides sensu strictu*, *C. nymphaeae*, and *C. godetiae* (Mosca et al., 2014; Talhinhas et al., 2005, 2009, 2011). Furthermore, in Portugal, novel *C. alienum* and *C. cigarro* have been recently reported as well in connection with OA (Cabral et al., 2024). The *Colletotrichum* genus is genetically and phenotypically extremely diverse and contains a growing number of species, presently aggregated into 11 species complexes/phylogenetic lineages (Jayawardena et al., 2016; Baroncelli et al., 2017; da Silva et al., 2020). *C. godetiae* and *C. nymphaeae* belong to the *C. acutatum* species complex, the largest one, which comprises 34 species (Zion Market Search, 2024). *C. gloeosporioides*, however, underwent a considerable re-classification, and many strains previously classified as such were re-classified as another, sometimes new species, and it has been assigned a species complex of its own (Jayawardena et al., 2016; Baroncelli et al., 2017; da Silva et al., 2020). Postharvest infections of fruits and vegetables by phytopathogenic fungi, including *Colletotrichum* species, are often controlled using antagonist microorganisms (Díaz et al., 2020; Sellitto et al., 2021). Some of the most popular of these are yeasts and yeast-based by-products (Hatoum et al., 2012; Freimoser et al., 2019; Lucas and Cássio, 2022). The control of pre-harvest fungal infections by biological control agents (Nigro et al., 2018), particularly by yeasts (Sellitto et al., 2021; Lucas and Cássio, 2022), is an increasingly promising alternative but has never been consistently and successfully used. Only a few commercial products using living yeasts are available (Sellitto et al., 2021).

The inhibition of fungal growth by yeasts occurs through different types of mechanisms. These include the secretion by the antagonist of volatile compounds (VOCs) toxic to the antagonized microbe (Contarino et al., 2019), or siderophores, mostly hydroxamate-type compounds (Zajc et al., 2019), which chelate ferric iron, promoting its transport into the yeast cell and in this way depleting the medium of this ion which is vital for the fungal cells. The antagonist yeast has often been described to also secrete lytic enzymes which are able to destroy fungal cell walls (*e.g.*, Liu et al., 2018; Giovati et al., 2021). These enzymes may be designated as mycocins or killer toxins although they are completely different from the virus-encoded classical killer proteins from *Saccharomyces cerevisiae* such as K28 (Schmitt and Tipper, 1990). These lytic enzymes/toxins include several types of chromosome-encoded glucanases, chitinases, or cellulases, which are able to destroy the cell walls or generate pores that indiscriminately permeate ions and small molecules, annulling life-supporting chemical gradients in the antagonized cell (Mannazzu et al., 2019). Additionally, yeasts in the context of antagonism have also been described, though much less often, to secrete reactive oxygen species (ROS), CO_2_, or even acids that lower the extracellular pH, specifically jeopardizing the survival of the fungi (Pretscher et al., 2018; Contarino et al., 2019; Druvefors et al., 2002; 2005).

The possibility that yeasts efficiently antagonize *Colletotrichum* species, particularly *C. gloeosporioides*, has been assessed before (Pesce et al., 2018; Peralta-Ruiz et al., 2023). *C. godetiae* and *C. nymphaeae* have never been, to the best of our knowledge, included in studies of yeasts as biocontrol agents. Strains of *S. cerevisiae* (Lopes et al., 2015; Campos-Martínez et al., 2016; Liu et al., 2018), *Meyerozyma guillermondii* (previously *Pichia guillermondii*) (Zhao et al., 2010), and *Pichia membranifaciens* (Belda et al., 2017), but more often *Wickerhamomyces anomalus* (previously *Pichia anomala*, *Hansenula anomala*, and *Candida pelliculo*sa) (*e.g.*, Pretscher et al., 2018; Lanhuang et al., 2022), stand out for their apparent extreme efficiency. Studies showed *W. anomalus* efficacy in controlling postharvest fungal pathologies such as blue mold decay in apples (Czarnecka et al., 2019; Zhao et al., 2021) and gray mold decay in tomatoes (Lanhuang et al., 2022; Zhu et al., 2023), highlighting its ability to inhibit pathogens’ spore germination and its ability to colonize fruits (Pretscher et al., 2018; Lanhuang et al., 2022; Zhu et al., 2023). Moreover, *W. anomalus* was reported to antagonize a series of other diverse fungal species, namely from the genus *Botryodiplodia* (Hashem and Alamri, 2009); *Botrytis* (Parafati et al., 2015; Lanhuang et al., 2022), *Monilia* (Czarnecka et al., 2019); *Curvularia, Fusarium*, and *Rhizoctonia* (Khunnamwong et al., 2020); *Moniliophthora* (Ferraz et al., 2021); *Aspergillus* and *Cladosporium* (Solairaj et al., 2020); *Penicillium* (Solairaj et al., 2020; Zhao et al., 2021); and *Alternaria* (Zhu et al., 2023). Importantly, *W. anomalus* was also reported to antagonize *C. gloeosporioides* originating, both yeast and fungal strains, from several sources (de Lima et al., 2012; Lima et al., 2013; Campos-Martínez et al., 2016; Zepeda-Giraud et al., 2016), including ripe olives (Pesce et al., 2018). Several of the above mechanisms were associated with the antagonistic activity of *W. anomalus*: (i) the secretion of killer toxins and hydrolytic enzymes (Friel et al., 2007; Parafati et al., 2017); (ii) the secretion of VOCs (Parafati et al., 2015; Oro et al., 2018; Contarino et al., 2019; Hoffmann et al., 2020); (iii) the production of high amounts of CO_2_ (Druvefors et al., 2002); (iv) the competition for nutrients and space; (v) the formation of biofilms; and (vi) mycoparasitism (Lima et al., 2013; Zepeda-Giraud et al., 2016; Pesce et al., 2018; Ferraz et al., 2021). Specifically, with regard to the specific antagonism of *C. gloeosporioides*, the secretion of VOCs (uncharacterized) and of large amounts of chitinase (Zepeda-Giraud et al., 2016) or β-1,3-glucanase (Lima et al., 2013), and the possible production of an exopolysaccharide-based biofilm (Zepeda-Giraud et al., 2016) have been referred to. Furthermore, SEM analysis of samples obtained in several *in vivo* or *in vitro* cultivation conditions showed the cells of *W. anomalus* extensively adhering to *C. gloeosporioides* hyphae, pulling them inwards and causing them severe damage (Lima et al., 2013; Zepeda-Giraud et al., 2016). Based on those observations, *W. anomalus* was suggested to act mainly as a mycoparasite. The same assumption was made by other authors based solely on this yeast’s ability to adhere to the fungal hyphae (Jijakli and Lepoivre, 1998; Hashem and Alamri, 2009).

In this study, 21 yeast strains originating from the olive biome and biotechnology fermentation-based industrial environments were used to test their capacity to antagonize the OA causal agents *in vitro*: *C. nymphaeae*, *C. godetiae* and *C. gloeosporioides s.s*. According to the results, one *Whickerhamomyces anomalus* strain stood out, LBMC 1105, which was isolated from *cachaça* distillation vats originating from spontaneous sugar cane fermentations (da Conceição et al., 2015) and previously identified as an extremely efficient BCA of cacao’s fungal phytopathogen *M. perniciosa* (Ferraz et al., 2021). The present study showed for the first time that besides *C. gloeosporioides*, *C. nymphaeae and C. godetiae* OA-causative fungi can be efficiently antagonized by yeasts, particularly by strains of *W. anomalus*. Additionally, this study also confirmed the LBMC 1105 strain has an exceptional ability to prey upon several taxonomically distant phytopathogenic fungi using a necrotrophic mycoparasitism mode of action. This raises an expectation that this yeast/strain can be implemented as a pre-harvest BCA against several fungal diseases in line with the urgent need for the development and implementation of safer control strategies for plant diseases.

## 2 Materials and methods

### 2.1 Microorganisms culture conditions

Yeast and filamentous fungi (**Table 1**) were equally maintained and cryopreserved in 30% glycerol at −80°C, and at 4°C on YPDA (10 g/L yeast extract, 10 g/L bacto peptone, 20 g/L D-glucose with 20 g/L agar) and MEA 2% (20 g/L Malt Extract, 20 g/L agar). Microbes were grown at 25°C in the same medium for 48–72 h prior to assaying, ensuring equally aged fresh inocula. Optimal growth conditions were established to measure the growth rates of fungi and yeasts on different media, at several pH, temperature, and aeration conditions (**Supplementary Table S1**). According to the results and the literature (Wharton and Diéguez-Uribeondo, 2004; Talhinhas et al., 2005), fungi and yeasts were equally grown at 25°C in MEA or in ME (20 g/L malt extract) (20 mL in glass tubes with Ø 3 cm and 13 cm height) at a pH of 5.5 with 200 rpm orbital shaking and a liquid/air ratio of 1:2.5. In liquid media, yeast cultures were followed spectrophotometrically at A_600 nm_, while fungal growth was visually inspected for the formation of a dense mycelium floating mass, which eventually filled most of the culture media and formed a ring on the glass tube walls. In solid media, the development of *Colletotrichum* mycelium was followed until it covered the whole plate.

**Table 1:** Microbial strains used in this work, their primitive origin, and their assigned code.

Yeast industrial strains			In this work
*Meyerozyma guillermondii*	LBMC 1015	Sugar cane-based production of *cachaça*	#1
*Saccharomyces cerevisiae*	LBMC 1025		#2
*Saccharomyces cerevisiae*	LBMC 1038		#3
*Saccharomyces cerevisiae*	LBMC 1096		#4
*Saccharomyces cerevisiae*	LBMC 1112		#5
*Saccharomyces cerevisiae*	LBMC 1113		#6
*Wickerhamomyces anomalus*	LBMC 1105		#7
*Saccharomyces cerevisiae*	FT280L CAT1	Sugar cane-based production of bioethanol	#8
*Saccharomyces cerevisiae*	FT134L PE2		#9
Yeast strains collected from infected orchards			
*Lachanceae thermotolerans*	PYCC 7205	Olives on tree	#10
*Kluyveromyces lactis*	PYCC 7201	Leaves on tree	#11
*Kodamaea ohmeri*	PYCC 7192	Soil under tree	#12
*Saccharomyces cerevisiae*	PYCC 8114	Olives on tree	#13
*Saccharomyces cerevisiae*	PYCC 8114	Olives on tree	#14
*Saccharomyces cerevisiae*	PYCC 8114	Olives on tree	#15
*Soliccocozyma phenolicus*	PYCC 7188	Olives on tree	#16
*Torulaspora delbrueckii*	PYCC 7193	Fallen leaves	#17
*Wickerhamomyces anomalus*	PYCC7203	Fallen ripe olives	#18
*Zygosaccharomyces bailii*	PYCC 7190	Fallen leaves	#19
*Zygosaccharomyces bailii*	PYCC 7197	Fallen ripe olives	#20
*Zygosaccharomyces rouxii*	PYCC 7198	Soil under tree	#21
Phytopathogenic fungal strains			
*Colletotrichum gloeosporioides s.s.*	CBS 100471	Olive orchard – Italy	–
*Colletotrichum godetiae*	ISA*	Olive orchard – Portugal	–
*Colletotrichum nymphaeae*	ISA*	Olive orchard – Portugal	–

LBMC: Molecular and Cellular Biology Laboratory, Federal University of Ouro Preto, MG, Brasil.

FT: Fermentec, Lda., SP, Brasil.

PYCC: Portuguese Yeast Culture Collection, UCIBIO, NOVA and Porto Universities, Portugal.

CBS: WI-KNAW Collections, The Netherlands.

ISA: School of Agriculture, University of Lisbon, Portugal. *These strains do not have an ascribed accession number (see Acknowledgements).

### 2.2 Evaluation of the antagonistic ability

Each combination of yeast/fungus was assayed in dual agar diffusion assays in MEA and in liquid co-cultures in ME, both at a pH of 5.5, as previously described (Ferraz et al., 2021). (i) MEA plates were kept at 25°C up to 8 days. The percentage of inhibition in solid media was determined by measuring the mycelium growth radius on opposite sides of the inoculum plug, away from (R1) and facing (R2) a yeast strike and quantified according to Royse and Ries (1978), as ((R1 − R2)/R1) × 100. (ii) Antagonism in liquid ME media was assessed by co-culturing a plug of actively growing mycelium, extracted with a sterile plastic cast to ensure approximately identical fungal biomass volume, with a suspension of yeast culture collected in the exponential phase at 1 × 10^6^ cells/ml. Co-cultures were incubated at the same temperature with orbital shaking at 200 rpm for 8 days at 25°C, and subsequently visually inspected and rated using an empirical 0–2 scale as previously described (Ferraz et al., 2021).

At the end of the incubation period in the liquid medium, the viability/death status of the remaining fungal cells was evaluated by staining with methylene blue (MB) and propidium iodide (PI). A small portion of the remaining mycelia was collected and washed with deionized water, and (i) a drop of MB 0.03% v/v was added. This was then incubated for 10 min at room temperature and observed under a light microscope (Olympus BX63F2 equipped with an Olympus DP74 camera), or (ii) placed in a microtube containing 500 μl PBS (Phosphate Buffered Saline) and 1 μl of PI (1 mg/ml) and incubated for 10 min in the dark at room temperature. Fluorescence was assessed with an epifluorescence microscope (Olympus BX63F2 equipped with an Olympus DP74 camera) using monochromatic light at 543 nm and an emission bandpass filter of 585–615 nm.

The putative secretion by yeasts of an agar-diffusible molecule or volatile compound was assessed in two manners. (i) Liquid ME yeast/fungus co-cultures after 8 days were decanted, and the liquid phase was centrifuged (9,500 rpm) and filtered (0.2 µm pore size), obtaining cell-free supernatants that were added to ME 2% at a 1:1 proportion and inoculated with a fresh mycelium plug for up to 2 weeks at 25°C. Two different controls were made. Ensuring the co-cultures supernatants did not retain any yeast cells, these were added to liquid ME tubes in a 1:1 proportion. Ensuring the fungi were not secreting any development-impeding compound themselves, identically obtained liquid ME fungus monocultures were decanted, centrifuged, inoculated with a fresh mycelium plug. Both types of control tubes were incubated for 2 weeks at 25°C. (ii) Septated MEA 2% Petri dishes were equidistantly inoculated with a mycelium plug and a yeast streak, allowing both cultures to share only the atmosphere inside the dishes. The plates were incubated for up to 2 weeks at 25°C. The areas covered by the mycelium were estimated using the Fiji Image J software (Schindelin et al., 2012).

### 2.3 Hydrolytic enzyme assays

Cell-free supernatants of 8-day-old fungus/yeast co-cultures in liquid medium were used to assay for the activity of chitinase, β-glucanase, and cellulase following protocols based on those of Lopes et al. (2015) and Hashem and Alamri (2009). Controls consisted of identical 8-day-old fungal cultures or overnight yeast cultures. The supernatants were obtained by decanting de growth medium in the case of the fungus-containing cultures, and by centrifuging the yeast-alone control cultures at 3000 rpm, at room temperature for 15 min. Supernatants were incubated with McIlvaine’s Buffer for 1 h at 50°C with specific substrates for each enzyme assay, i.e., colloidal chitin (10 mg/ml), carboxymethyl cellulose (CMC) (0.55%), and laminarin (4 mg/ml) to assay for the activity of chitinase, cellulase, and β-glucanase, respectively. The quantification of the resulting reduced sugars was done using the colorimetric assay with DNS (3,5-dinitrosalicylic acid) (Miller, 1959). Calibration curves for estimating the amounts of reducing sugars were obtained using glucose and N-acetyl-D-glucosamine. Negative controls were done using reaction mixtures without any enzyme substrate.

### 2.4 Blue agar Chrome Azurol S assay for siderophore production detection

A Chrome Azurol S (CAS) assay was performed mostly using the procedure originating from Schwyn and Neilands (1987) and protocoled by Louden et al. (2011), as well as that of Nally et al. (2015), with some modifications. Glucose-agar base medium was prepared by mixing glucose (20 g/L) and agar (20 g/L) with piperazine-N,N’-bis(2-ethanesulfonic acid) (PIPES) solution (32.24 g/L) at a pH of 6.8. Blue dye mixture was prepared by sequentially and slowly mixing three solutions under manual stirring: (i) FeCl_3_•6H_2_O (1 mM in 10 mM HCl), (ii) CAS (60.5 mg/L), and (iii) HDTMA (hexadecyltrimethylammonium bromide) (72.9 mg/L) at a proportion of 1:5:4. Both glucose-agar base and BD mixture were autoclaved, allowed to cool to approximately 50°C, and added to YNB w/amino acids (6.7 g/L) (Nally et al., 2015) at a proportion of 8:1:1.

### 2.5 Scanning electron microscopy

Samples of 8-day-old liquid medium-grown fungi or fungi and yeasts, and controls of overnight yeast cultures were used. Yeast-alone overnight cultures were centrifuged at 3000 rpm at room temperature for 15 min and the supernatant was discarded. Fungi-containing cultures were first decanted, and the fungal biomass was repeatedly washed in distilled water, and gently shaken until the rinsed water was clear. The biomass in the plug was then separated from the remaining agar by gently peeling the mycelium with sterile tweezers. Sample fixation, dehydration, and coating were performed as previously described (Ferraz et al., 2021). Observations were done as before, with a NanoSEM FEI Nova 200 at a 5/10 kV and a through-lens detector (TLD) at the SEMAT Unit at the University of Minho (http://www.semat.lab.uminho.pt). Yeast cells were measured using the Fiji Image J software (Schindelin et al., 2012) to determine the length of the longer and the perpendicular shorter axis of each cell in light microscopy or SEM images. Cell volume was estimated applying the formula for the volume of a oblate ellipsoid: vol = 4/3.П.a^2^.b (a being the shorter axis). Using the same micrographs, the volume of the spheroid/ellipsoid structures inside the hyphae were estimated using the same formula.

### 2.6 Statistical analysis

All the results correspond to at least three independent assays, each with three replicates. The results from enzyme assays and SEM measurements were treated with a one-way analysis of variance (ANOVA) and t-test, respectively. The assumptions of normality and homogeneity of variances were checked visually by inspecting the scatterplots of the residuals. *Post hoc* multiple comparison tests were carried out using Tukey’s Honest Significant Difference (HSD) method to identify significant differences (*p-value* < 0.05).

## 3 Results and discussion

The yeast strains used in the present work (**Table 1**) originate from two distinct types of environments. Some were primordially isolated from OA-infected olive orchards (PYCC strains) and others are used in biotechnology industries (LBMC and FT strains). The reason underlying the choice of the first group was the possibility that the yeasts sharing the same microenvironmental niches as the fungi might have acquired specific antagonism abilities. This strategy was successfully previously used to isolate *S. cerevisiae* strains from wine (Liu et al., 2018) and *W. anomalus* strains from avocado fruits (Campos-Martínez et al., 2016) and olive trees (Pesce et al., 2018), all of which originated from anthracnose infected vineyards and orchards. The second group of yeasts was chosen because they are strains that are extremely resilient to diverse stressful environmental conditions (da Conceição et al., 2015). Some of them are freely exploited commercially for industrial fermentations (Cerlev, Lda (https://www.cerlev.com.br/) and Fermentec, Lda. (https://www.fermentec.com.br)), which may facilitate their potential pre-harvest implementation. This group includes *W. anomalus* #7, an exceptionally stress-resistant yeast (da Conceição et al., 2015) that was previously described to strongly antagonize the causative agent of Witches’ Broom disease in cacao, *Moniliophthora perniciosa* (Ferraz et al., 2021). However, the *Colletotrichum* strains that were used (**Table 1**) were primordially collected from infected olive orchards in Italy (CBS strain) and in Portugal (ISA strains). These isolates corresponded to three different species, *C. gloeosporioides, C. godetiae*, and *C. nymphaeae*, which are more often associated with the onset and development of olive anthracnose (Mosca et al., 2014; Talhinhas et al., 2005, 2009, 2011; Cabral et al., 2024).

### 3.1 Yeasts antagonizing *Colletotrichum* species

In the present work, antagonism was first assessed using dual agar diffusion assays combining all the yeasts and fungal species outlined in **Table 1**. These tests were performed using uniformized culture conditions established according to the preliminarily determined growth rates of fungi (**Supplementary Figure S1**) bearing in mind specificities for *Colletotrichum* cultivation parameters (Talhinhas et al., 2005). The results showed that all the yeasts were able to antagonize to some extent the three *Colletotrichum* species (**Table 2**). The strongest effect was observed with the two strains of *W. anomalus*, one of which is the aforementioned *W. anomalus* #7 (Ferraz et al., 2021). Concurringly, in the literature, other isolates from *W. anomalus* were reported to act as strong antagonists of *C. gloeosporioides* and *C. acutatum*, reducing the development of the mycelium by ≥60% (Zepeda-Giraud et al., 2016) or ≥87% (Campos-Martínez et al., 2016), and the severity of the disease by ±60% (Pesce et al., 2018), or ≥70% (Campos-Martínez et al., 2016).

**Table 2:** Degree of antagonism between yeast strains in **Table 1** and phytopathogenic *Colletotrichum* species.

	Yeast industrial strains										
	#1	#2	#3	#4	#5	#6	#7	#8	#9	#10	
*C. gloeosporioides* s.s	3.2 ± 5.5	17.5 ± 7.3	19.1 ± 4.8	25.4 ± 15.3	46.0 ± 22.5	23.8 ± 4.8	75.7 ± 7.4	57.1 ± 24.7	19.1 ± 4.8	22.2 ± 5.5	
*C. godetiae*	40.9 ± 7.9	22.7 ± 25.3	33.3 ± 26.6	25.8 ± 9.5	21.2 ± 11.4	59.1 ± 4.6	28.8 ± 9.5	10.6 ± 14.6	9.1 ± 4.6	41.9 ± 7.3	
*C. nymphaeae*	25.9 ± 3.6	33.3 ± 24.7	47.2 ± 17.5	54.7 ± 2.1	51.2 ± 22.8	25.3 ± 17.1	62.9 ± 7.6	37.9 ± 20.3	14.6 ± 12.4	49.1 ± 4.3	
	Yeast strains collected from infected orchards										
	#11	#12	#13	#14	#15	#16	#17	#18	#19	#20	#21
*C. gloeosporioides* s.s	33.3 ± 35.9	22.2 ± 22.5	28.6 ± 0.0	25.4 ± 24.0	28.6 ± 4.8	0.0 ± 0.0	31.8 ± 28.7	70.8 ± 7.2	28.6 ± 31.2	11.1 ± 7.3	14.3 ± 12.6
*C. godetiae*	55.2 ± 18.9	38.1 ± 5.7	44.1 ± 10.6	52.2 ± 10.6	55.9 ± 16.0	43.2 ± 18.4	32.1 ± 27.8	70.5 ± 0.8	46.1 ± 17.9	33.3 ± 11.6	48.2 ± 5.6
*C. nymphaeae*	52.8 ± 14.1	56.3 ± 5.5	61.3 ± 5.2	62.2 ± 7.9	65.4 ± 3.8	30.2 ± 2.8	65.9 ± 3.0	74.5 ± 4.3	55.7 ± 3.1	54.0 ± 9.7	43.6 ± 19.4

Results are the percentage of inhibition in MEA, according to Royse and Ries (1978). Values are average across n≥3 independent assays, each with three identical replicates, and standard deviation, after 8 days of incubation at 25°C. Shaded cells show the highest inhibition.

**Figure 1:**
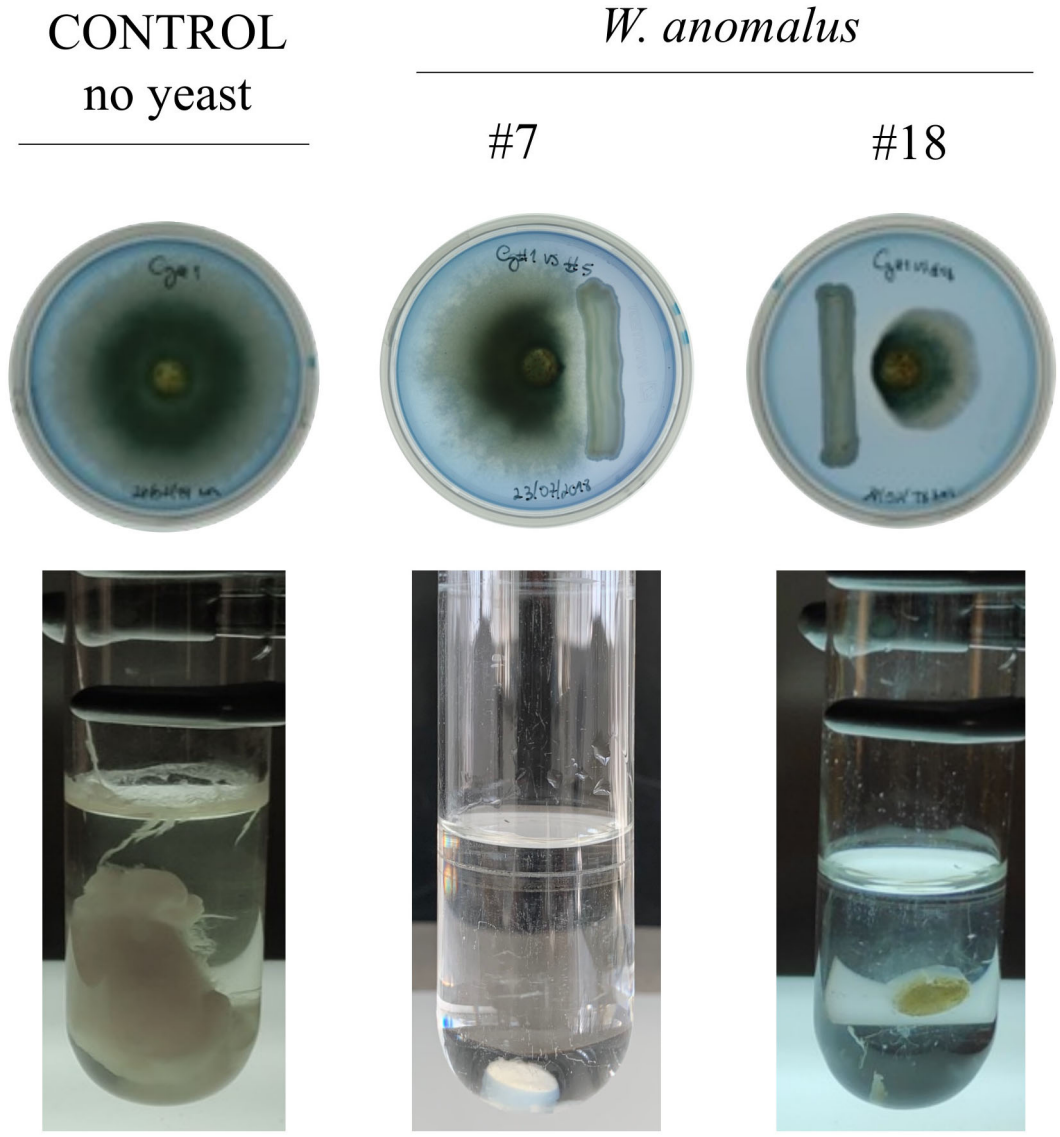
*W. anomalus* #7 and #18*/C*. *gloeosporioides* antagonism assays in solid (MEA) and liquid (ME) media. The results after 8 days at 25°C showed a clear inhibition of fungal mycelia development, stronger in liquid media yeast-fungus co-cultures.

Antagonism has been previously shown to be better assessed by co-incubating the yeasts with the fungi in liquid medium (Ferraz et al., 2021), allowing any antifungal metabolite that might be responsible for the antagonism a more efficient diffusion and contact with the mycelium. Moreover, it also allows for more efficient nourishment of the yeast and fungal cells as the cultures stay alive and metabolically active for longer periods of time. Importantly, liquid co-culture also promotes physical proximity and contact between antagonist and antagonized cells, which may be necessary to trigger and/or develop efficient antagonism (Ferraz et al., 2021). The above yeast/fungus combinations were therefore assayed in liquid medium according to the methodology previously developed (Ferraz et al., 2021). The results were noted after 8 days of co-culturing. As expected, the antagonism was globally stronger (**Supplementary Table S1**). *W. anomalus* strains #7 and #18 maintained their high antagonism ability against all three fungal species (**Figure 1**).

**Figure 2:**
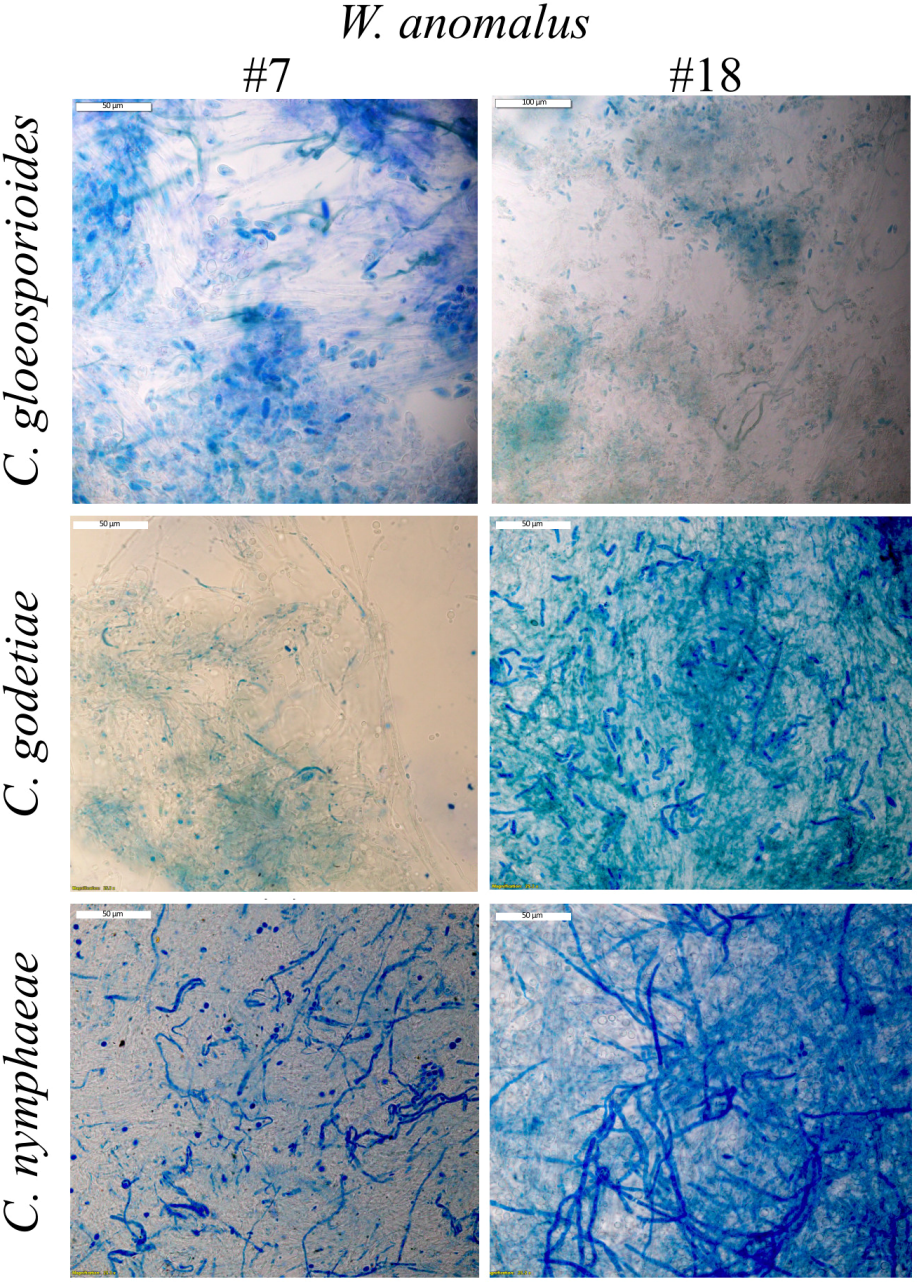
Light microscopy observation of *Colletotrichum* mycelia stained with MB, after being in contact with *W. anomalus* #7 and #18 for 8 days at 25°C. Staining reveals fungal cell death.

The absence of mycelium development in co-cultures can derive from the complete death of the fungal cells (cytocide effect) or just their inhibition from multiplying (cytostatic effect), hence the need to determine the viability of the remaining mycelium. This was done by staining samples of fungi, after 8 days in co-culture with the yeasts, with methylene blue (MB). Extensive mycelia staining was observed (**Figure 2**). Additionally, each remaining mycelium was transferred to fresh growth medium. The results showed that the mycelium from all yeast/fungus combinations was able to resume growth (not shown), indicating that, despite the extensive MB and PI staining observed corresponding to extensive cell death, some part of the mycelium was still alive and able to grow. These results suggested that the proportion of the number of yeast and fungal cells might be crucial for obtaining the total death of the fungus. Several authors refer to the need for a specific concentration of *W. anomalus* yeast cells (10^7^ or 10^8^ cells/mL) so that fungal lesions caused by fruit and tomato decay fungi can be completely avoided, both *in vitro* and *in vivo* (Liu et al., 2018; Zhao et al., 2021; Lanhuang et al., 2022; Zhu et al., 2023). Thus, we used 10^6^ cells/mL. Moreover, from the point of view of the yeast’s lifespan, the 8-day incubation period used to obtain the co-culture results is rather long from the yeast culture point of view and could affect their viability. If a considerable part of the yeast population dies during incubation, a disproportion between the number of living yeast and fungal cells is expected, which could negatively impact our results. Accordingly, it was reported that if fruits were inoculated with the same amount of *W. anomalus* or *M. guillermondii* before instead of after infection with *C. gloeosporioides*, their protection against disease spreading was considerably higher, indicating the need for most yeasts to be young and metabolically active when interacting with the fungus de Lima et al. (2012). In this work, identically to what was done with the fungi, the samples of yeast populations from 8-day co-cultures were centrifuged, washed, and re-inoculated in fresh medium, where they regrew abundantly (not shown).

### 3.2 VOCs, hydrolytic enzymes, and siderophores are not specific to *W. anomalus* antagonism

Bearing in mind all the above results, the study focused on assessing the mode of action of *W. anomalus* #7 and #18, the strongest *C. gloeosporioides, C. godetiae*, and *C. nymphaeae* antagonists from the original 21 yeasts. *W. anomalus* was previously reported to antagonize other microorganisms by secreting β-glucanase, and several volatile compounds, most often ethyl acetate (Lucas and Cássio, 2022), ethanol, and CO_2_ (Druvefors et al., 2002; Contarino et al., 2019), as well as other less often reported strategies, such as the competition for nutrients and space (Zhao et al., 2021; Pesce et al., 2018). These mechanisms have the potential to obstruct *Colletotrichum* from growing. Although they may be individually or synergistically involved in the antagonistic process, it is possible that they only indirectly help the yeast cells to ensure global supremacy over fungi during the cultures’ contact (Passoth et al., 2006; Pretscher et al., 2018; Tilocca et al., 2020; Lucas and Cássio, 2022). Accordingly, the disruption of the β-glucanase-encoding *WaEXG1* and *WaEXG2* did not impair the yeast’s ability to act as BCAs for several filamentous fungi (Jijakli and Lepoivre, 1998; Grevesse et al., 2003; Friel et al., 2007). These genes’ expression responds *in vitro* to the fungal cell walls, though an identical stimulation was not observed *in vivo* in wound-infected fruits (Parafati et al., 2017). Importantly, *W. anomalus* was reported to act as a mycoparasite, mostly based on the fact that the yeast cells adhered to the hyphae which were eventually emptied (Hashem and Alamri, 2009; Ferraz et al., 2021).

**Figure 3:**
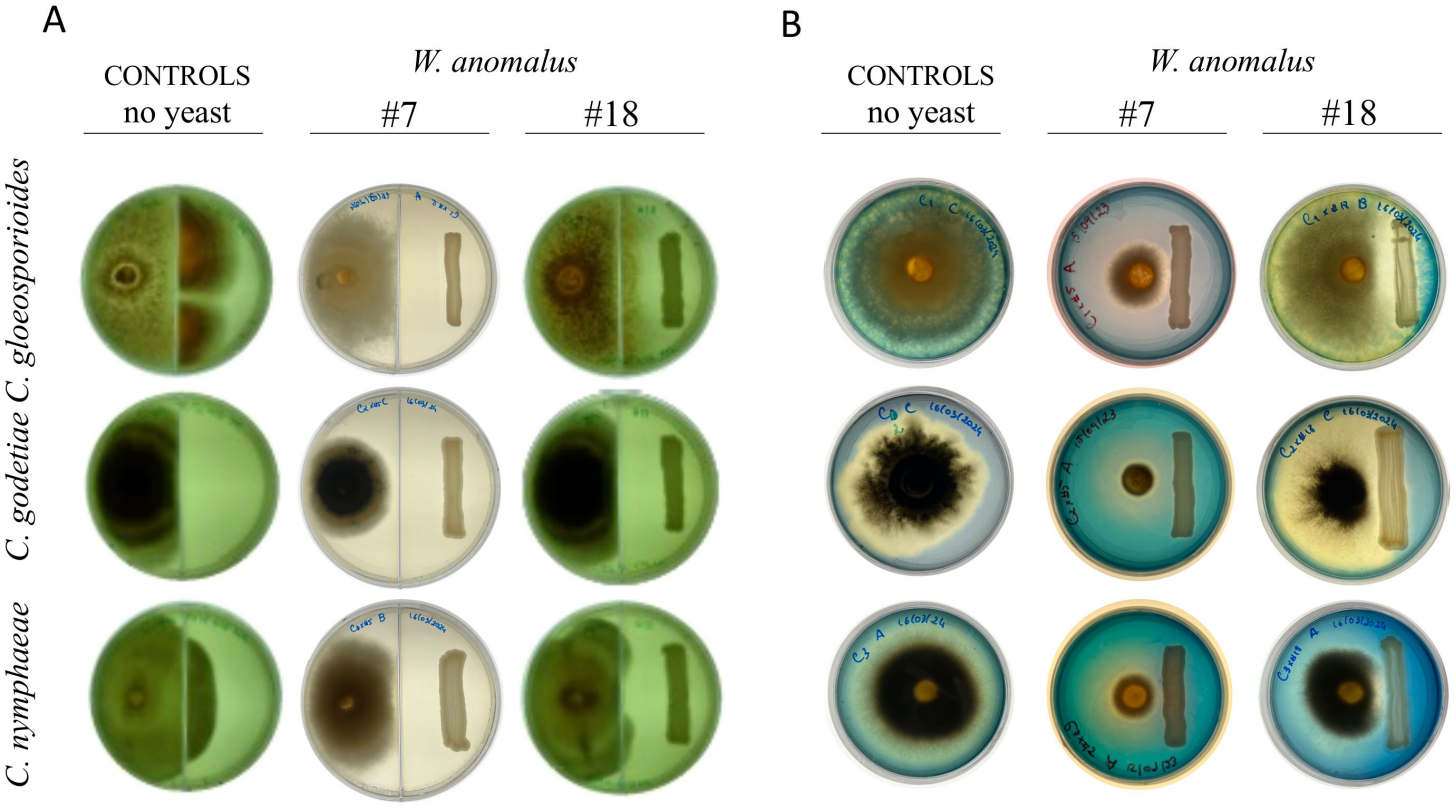
Antagonism of *W. anomalus* #7 and #18 against the three *Colletotrichum* species. **(A)** Yeasts and fungi were inoculated in MEA, in opposite parts of septated Petri dishes. The results after 8 days at 25°C showed that the fungi grew well, with only a non-statistically significant reduction compared to the no-yeast control cultures, suggesting that antagonism did not depend on VOCs produced by the yeasts. **(B)** CAS assay was applied to yeast-fungi co-cultures to detect the putative production of siderophores. The results after 8 days at 25°C showed that instead of the yeasts, the three fungal *Colletotrichum* species secreted siderophores to different degrees.

In this work, *W. anomalus* #7 and #18 were assessed against *C. gloeosporioides, C. godetiae*, and *C. nymphaeae* with regard to the secretion of VOCs or CO_2_, by repeating the antagonism tests on solid media using septated Petri dishes inoculated with the mycelial plug and the yeast streak on each side of the septum. These dishes allowed the two microbes to share the atmosphere inside the dish without ever contacting each other or sharing the growth medium. The results (**Figure 3A**) were described after 8 days. In the presence of *W. anomalus* #18, *C. gloeosporioides* and *C. nymphaeae* filled their side of the plate and outgrew the septum, while *C. godetiae* still grew abundantly although it did not overgrow the septum. Otherwise, in the presence of *W. anomalus* #7, all three fungi grew to a lesser extent, which could indicate that #7 produced some volatile compound in amounts that were only able to retard the fungal development but not fully impede it. To verify this, the areas occupied by the mycelium in the controls and in the assays with yeast were estimated and compared. No statistically significant differences were observed (*p-values* > 0.05 (n=9 for each fungus)) (not shown). These results suggested that the inhibition of fungal growth by either of these yeast strains did not depend on the secretion of a volatile compound.

Other strains of *W. anomalus* were shown to produce considerable amounts of ethyl acetate and CO_2_ (Druvefors et al., 2002; Masoud et al., 2005; Parafati et al., 2015; Oro et al., 2018; Contarino et al., 2019; Hoffmann et al., 2020), which were associated with antagonism against a very large variety of food decay microbes and phytopathogens (Fredlund et al., 2004; Masoud et al., 2005; Oro et al., 2018; Contarino et al., 2019; Hoffmann et al., 2020). *W. anomalus* was also reported to secrete VOCs against *Penicillium roqueforti* (Druvefors and Schürer, 2005) and *C. gloeosporioides* (Zepeda-Giraud et al., 2016). One cannot disregard that the production of these or other compounds is not only strain-dependent but also varies with environmental conditions and the life cycle of the yeasts and the fungi, raising questions about their actual role in or need for antagonism to occur (Tilocca et al., 2020).

If instead of volatile compounds, some other molecule would be secreted by *W. anomalus*, it would be effective in cultures freely sharing the agar or liquid medium. *W. anomalus* has repeatedly been reported to secrete lytic enzymes (glucanases and chitinase), although only once was it reported to secrete siderophores (Khunnamwong et al., 2020). Thus, cell-free supernatants from mature liquid co-cultures were used to inoculate fresh mycelium. These supernatants were filtered to avoid the presence of cells but were not autoclaved, to allow any putative enzyme or chemical compound to remain stable. The three *Colletotrichum* species grew abundantly (not shown), identical to the controls, ruling out the possibility that either *W. anomalus* #7 or *W. anomalus* #18 secrete compounds/molecules able to harm the fungal cells. These results were identical to those obtained by de Lima et al. (2012), who showed that *W. anomalus* cultures, either filtered or autoclaved, lost completely the ability to inhibit the germination of *C. gloeosporioides* conidia. Agar-diffusible compounds include siderophores and enzymes. The secretion of siderophores can be detected by growing the microbes in plates containing Chrome Azurol S (CAS assay) (Schwyn and Neilands, 1987; Neilands, 1993). Once more, the *Colletotrichum* species were tested in this way against *W. anomalus* #7 and *W. anomalus* #18. The results showed that neither strain produced siderophores (**Figure 3B**). In contrast, the three *Colletotrichum* species produced siderophores to some extent when cultivated alone. *C. godetiae* produced a more accentuated effect, followed by *C. nymphaeae* and *C. gloeosporioides*. In no case was the growth of the yeast affected. Still, the differences between the results obtained with *W. anomalus* #7 and #18 suggest that the inability of the fungi to properly grow in the presence of the #7 strain precluded their siderophore production to levels that can be detected. Otherwise, the same fungi growing alone or in the presence of #18, which does not inhibit growth as strongly as #7, produced a significant amount of siderophores. Many filamentous fungi were previously reported to secrete siderophores (Baakza et al., 2004; Winkelmann, 2007), including *C. gloeosporioides*, which was shown to secrete ferricrocin, a phytotoxic siderophore, apparently without any specific cultivation induction requirement (Ohra et al., 1995).

The secretion of hydrolytic enzymes by *W. anomalus* #7 was tested using supernatants from mature yeast-fungus co-cultures in liquid medium. According to the literature, *W. anomalus* secretes several hydrolytic enzymes, such as β-1,3-glucanase (Jijakli and Lepoivre, 1998; Grevesse et al., 2003; Izgü and Altinbay, 2004; Izgü et al., 2007; Friel et al., 2007; Lima et al., 2013; Schwentke et al., 2014; Hong et al., 2017; Pretscher et al., 2018), chitinase, cellulase or protease, and, to a lesser extent, amylase and β-glucosidase (Pretscher et al., 2018). Authors diverge when considering that these enzymes do have a direct role in *W. anomalus* antagonism because the attempts to demonstrate *in vitro* their direct effect on antagonism failed (Pretscher et al., 2018). This is because enzymes and their quantity/activity vary considerably from strain to strain and because the efficient lysis of fungal cell walls would require the simultaneous synergistic action of various enzymes (Salazar and Asenjo, 2007). The supernatants of both *W. anomalus* #7 co-cultured with any of the three *Colletotrichum* species showed activity for β-glucanase and chitinase (**Supplementary Figure S2**). However, the activity of β-glucanase was not significantly different in supernatants deriving from any of the yeast-fungus co-cultures or from the yeast-alone controls (F(5.48) = 0.457, *p* = 0.806). The same occurred with the activity of chitinase in the co-cultures with *C. gloeosporioides* or *C. nymphaeae*. Only *W. anomalus/C. godetiae* co-cultures displayed chitinase activity significantly higher than that of the control cultures (F(5.48) = 3.814, *p* = 0.0055) (**Supplementary Figure S2**). The structure and relative concentrations of the polysaccharides from the cell wall of filamentous fungi are mostly unknown, though it is acknowledged that it varies considerably with the morphotype. There is no information available as to the specific chemical composition of the cell walls of these *Colletotrichum* species. The only information available is that β-(1,3) and β-(1,6) glucans are absent in the biotrophic hyphae of *C. graminicola* (Gow et al., 2017). Therefore, one can only speculate that *C. godetiae* cell walls may be richer in chitin than *C. gloeosporioides* or *C. nymphaeae*.

All considered, the results suggested that the *W. anomalus* #7 and #18 strains did not secrete VOCs, CO_2_, or siderophores in amounts high enough to be detected or harmful, but *W. anomalus* #7 secretes β-glucanase and chitinase, although only the secretion of this last enzyme could be associated with the antagonism of *C. godetiae.* Nevertheless, some agar-diffusible compounds/molecules must be secreted by *W. anomalus* #7 and *W. anomalus* #18 to justify the fungal growth inhibition of the yeast colonies observed in the agar assays (**Figure 1**; **Table 2**). *W. anomalus* was shown to secrete non-enzymatic killer toxins (de Ingeniis et al., 2009; Farkas et al., 2012) although their involvement in this yeast’s mode of action against fungi has not been explored. However, true extensive killing of mycelium, as observed in liquid cultures stained with MB (**Figure 2**), did not occur in the agar plates with MB (not shown), suggesting it required actual physical contact between the yeast and fungal cells, as previously described for *W. anomalus* #7 against *Monilliophthora perniciosa*, the causative agent of the Witches’ broom disease of the cacao plant (Ferraz et al., 2021), or for another strain of this yeast against *Botryodiploidia theobromae* (Hashem and Alamri, 2009). *W. anomalus* may have the same antagonism strategy against all phytopathogens even if it differs in efficiency and strength, or it may also be that the yeast is able to discriminate between different fungi and adopt different antagonism strategies. The results above showed that *W. anomalus* did not behave in exactly the same fashion in the presence of the three *Colletotrichum* species, since *W. anomalus* co-cultured with *C. godetiae* secreted higher amounts of chitinase. To investigate this possibility, liquid medium yeast-fungus co-cultures were assessed microscopically. *W. anomalus* #7 was chosen for this purpose.

### 3.3 Microscopy assessment of yeast–fungus co-cultures

**Figure 4:**
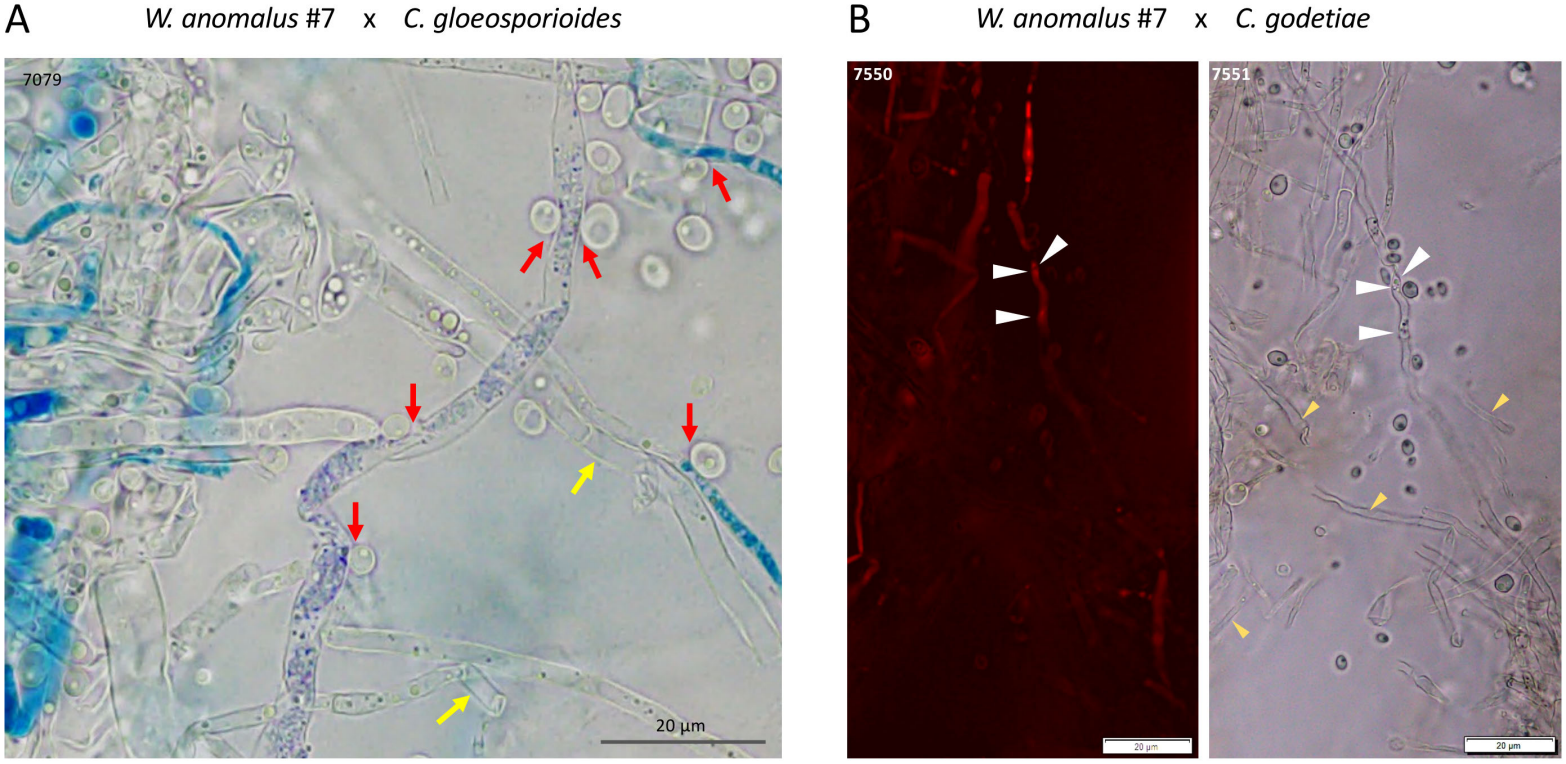
Light microscope micrographs of *W. anomalus* #7 co-cultured with *C. gloeosporioides* for 8 days in ME at 25°C, stained with MB **(A)** and PI **(B)**. **(A)** Red arrows indicate yeasts pulling the hyphae inwards, and yellow arrow shows empty hyphae. **(B)** White arrows show hyphae constricted by yeasts where small areas of PI-stained cell contents can still be seen, whilst many empty hyphae are also found (yellow arrows).

**Figure 5:**
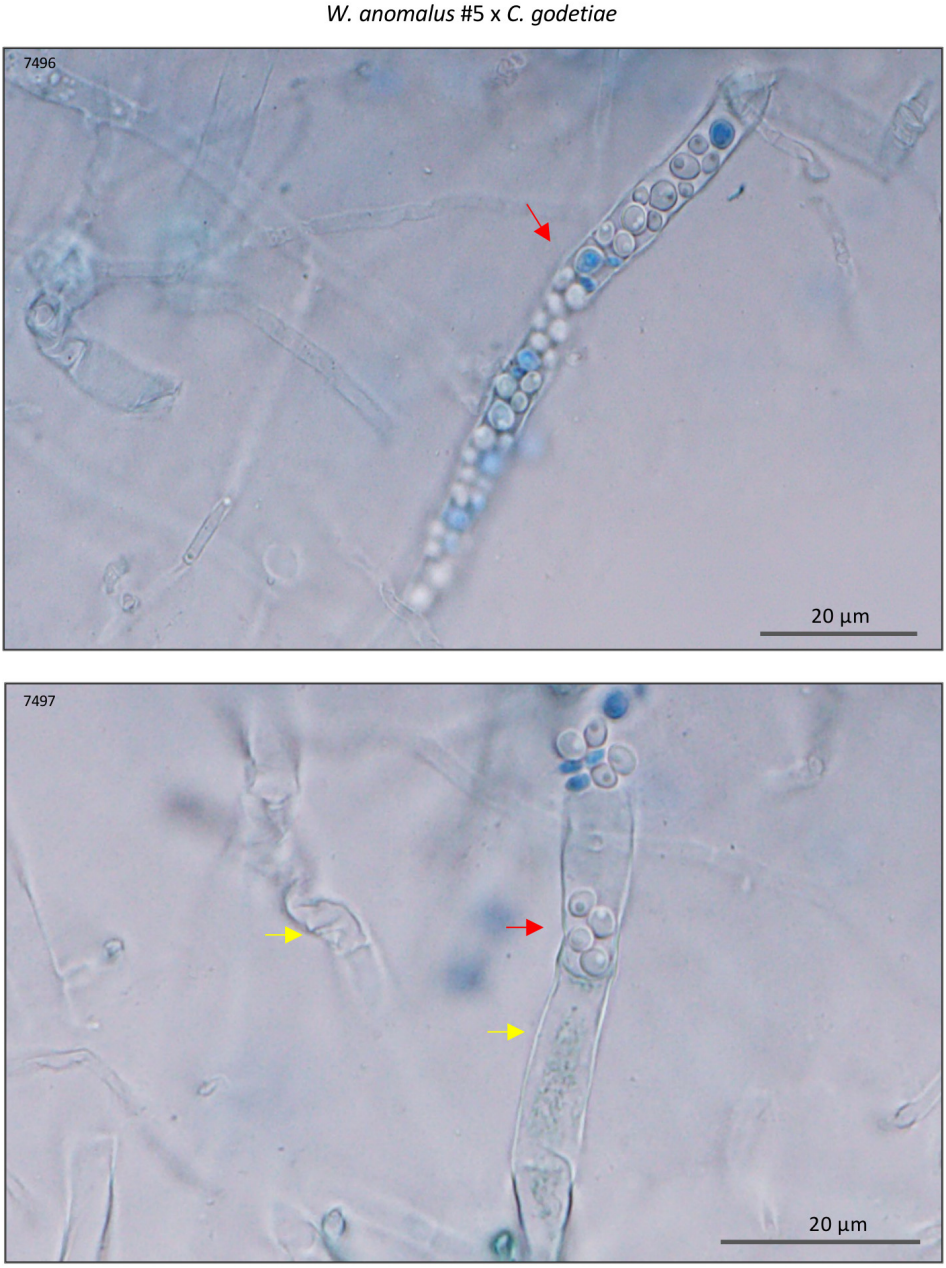
Light microscope micrographs of MB-stained samples of *W. anomalus* #7/*C. godetiae* showing yeasts packing inside empty hyphae (red arrows) and empty or emptying/emptied hyphae (yellow arrows). Results were observed after 8 days incubation at 25°C.

Samples of 8-day-old yeast–fungus co-cultures were first stained with MB and scanned under light microscopy. It was possible to see *W. anomalus* #7 cells adhering to hyphae and appearing to pull them inwards, as well as large amounts of empty hyphae (exemplified in **Figure 4A**). When analyzing the clear field microscope images in comparison with their PI-stained counterparts, it was evident that much of the mycelium that was not stained with PI were empty hyphae. Only the small remaining pieces of cytoplasm in the partially emptied hyphae were stained (illustrated in **Figure 4B**). These observations showed that *W. anomalus* #7, identically to that previously reported with *M. perniciosa*, adheres to the *C. gloeosporioides, C. godetiae*, and *C. nymphaeae* hyphae, pulling them inwards and draining them, causing the hyphae to shrink and eventually collapse. This behavior is identical to what was reported to occur with *C. gloeosporioides* when facing a different *W. anomalus* strain (Lima et al., 2013; Zepeda-Giraud et al., 2016), and with the same *W. anomalus* #7 strain facing a different fungus, *M. perniciosa* (Ferraz et al., 2021). Most authors consider that the simple adhesion of yeast cells to the hyphae is a reliable indication of mycoparasitism. Nevertheless, the very definition of parasitism demands that one of the organisms takes advantage of the other, which may eventually die. The abundance of empty hyphae in the co-cultures could indicate such a mechanism, with yeasts preying on the fungal cells and feeding on their contents as contact or invasive necrotrophic mycoparasites (Jeffries, 1995). That would be consistent with the unexpected vitality of the yeast cultures observed after being co-cultured for 8 days with the fungi when the medium was depleted of major nutrients. Thus, for the first time, numerous yeasts were found packed inside *C. godetieae* hyphae (**Figure 5**). The cell volume of *W. anomalus* #7 inside the hyphae, estimated from micrographs, was in fact significantly different when compared with planktonic cells in the same cultures’ supernatants [respectively, 12.2 ± 6.1 µm^3^ (n=32) and 26.1 ± 20.1 µm^3^ (n=42)] (*p-value* < 0.001). However, the estimated value of the smallest planktonic yeast cells was similar to that of yeasts found inside the hyphae. This is the first indication of true yeast predation and is consistent with invasive necrotrophic mycoparasitism. Identical yeast-invaded hyphae were not observed in co-cultures with *C. gloeosporioides* and *C. nymphaeae*, which, however, showed extensive emptied hyphae. Doubt remained as to whether *W. anomalus* may act differently towards these fungi, possibly as a contact mycoparasite that kills the fungus without penetrating it. To try and clarify this issue, SEM analysis of samples from the 8-day co-cultures was performed.

**Figure 6:**
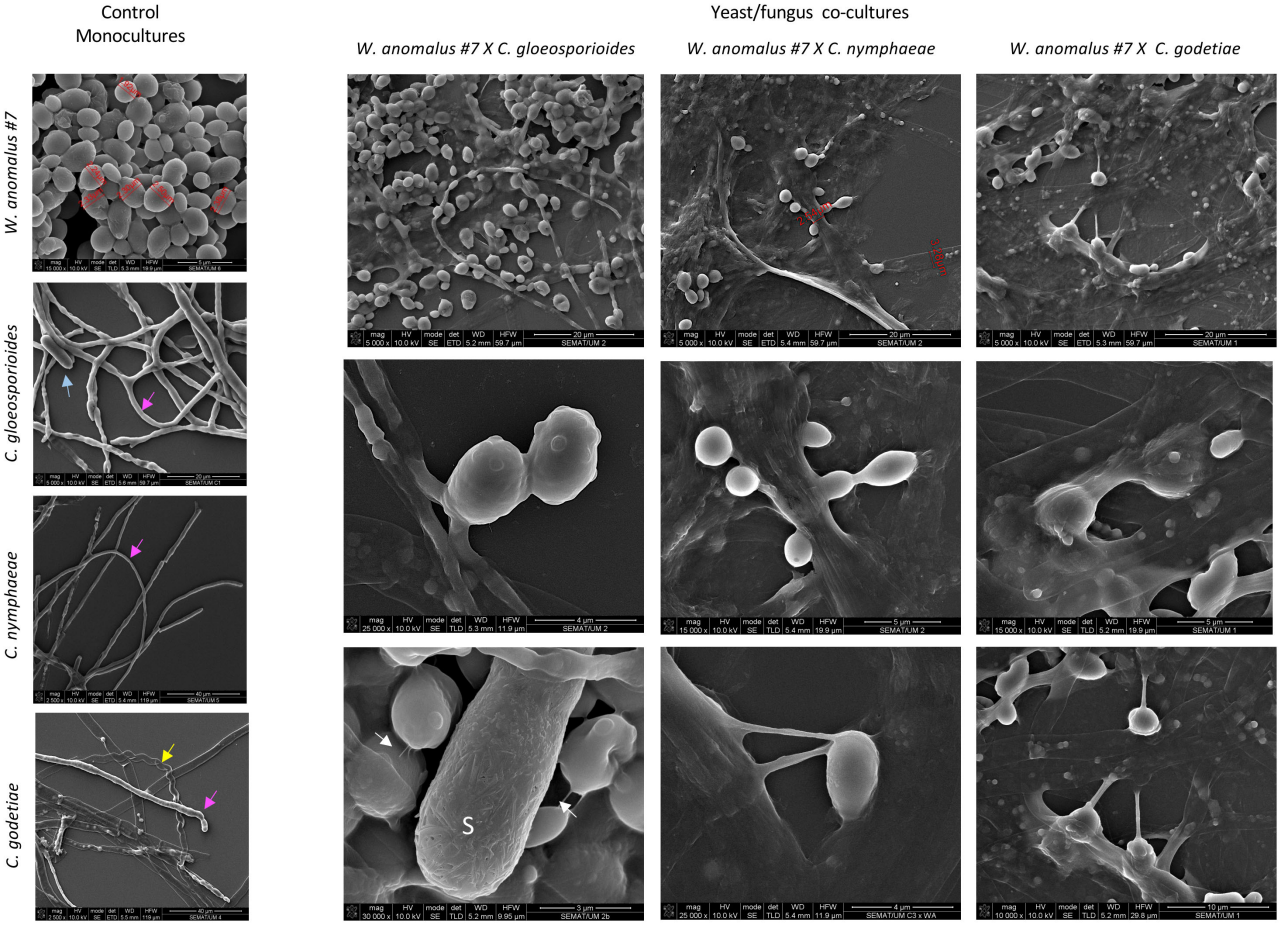
SEM micrographs depicting the *W. anomalous* #7 and the *Colletotrichum* fungi cultivated alone or in combination. Samples were observed after co-cultivation for 8 days in liquid ME at 25°C. Arrows: pink – regular healthy hyphae; yellow – empty curled hyphae; blue: fungal spore (S). Upper panels of co-culture results show mucilage covering the cells and extensive adhesion of yeast cells to the hyphae. Middle panels show yeasts fused with hyphae. Lower panels show the mucilage creating long and thick bridges between yeast cells and between yeast and fungal cells, and fimbriae connecting yeast cells in the case of *C. gloeosporioides* (white arrows).

The observations with SEM also revealed extensive yeast adhesion to the hyphae of the three *Colletotrichum* species, the concave pressure yeast cells made against their hyphae, and their fusion (**Figure 6**). Subsequent hyphal draining is implied from the finding of a large number of empty hyphae, identical to what was observed with light microscopy. The ability of *W. anomalus* cells to adhere to and fuse with hyphae has been described to require the production and secretion of a viscous biofilm-like extracellular matrix (ECM) polysaccharide (Hashem and Alamri, 2009; Ferraz et al., 2021). Accordingly, this yeast can form biofilms *in vitro* (Zepeda-Giraud et al., 2016; Khunnamwong et al., 2020). The formation of biofilms was recently added to the list of requirements for a microbe to act as a BCA (Sipiczki, 2023; Peralta-Ruiz et al., 2023). However, this feature remains controversial since yeast pseudohyphae developed for biofilm formation could become harmful to plant tissue (Ma et al., 2023). Furthermore, Wisniewski et al. (1991) clearly showed that the adhesion of *Pichia guillermondii* to the hyphae of *Botrytis cinerea* displayed properties of a lectin-mediated bond. However, this was not further explored.

**Figure 7:**
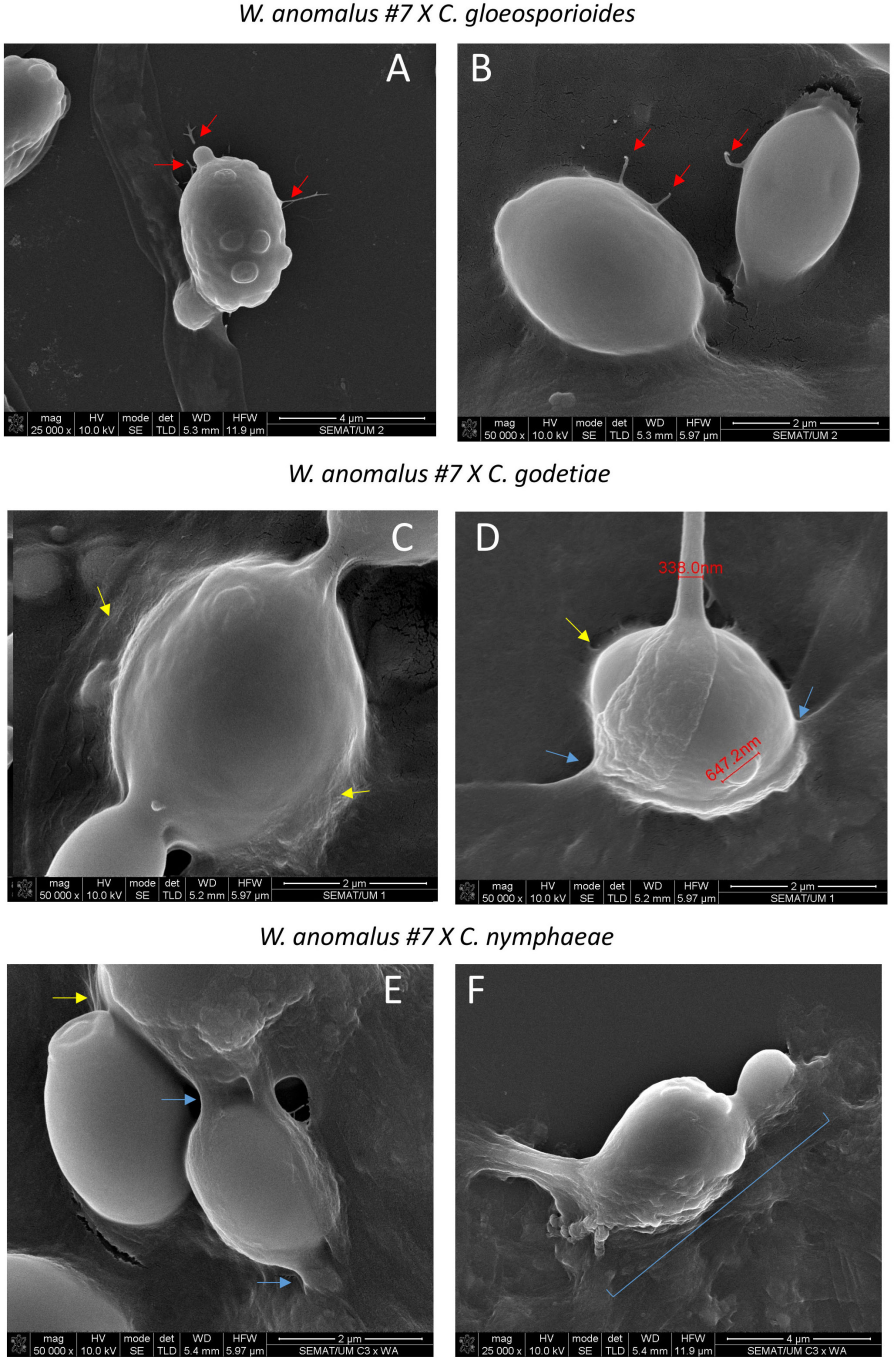
SEM micrographs of yeast/fungus co-cultures exemplifying details of (i) the fimbriae found connecting yeast cells (red arrows) **(A, B)**; (ii) the mucilage/ECM covering both the yeast and fungal cells (yellow arrows) **(C–E)**; (iii) the complete fusion of the yeast with the hyphal wall on the way to penetration (blue arrows and brace) **(D–F)**.

**Figure 8:**
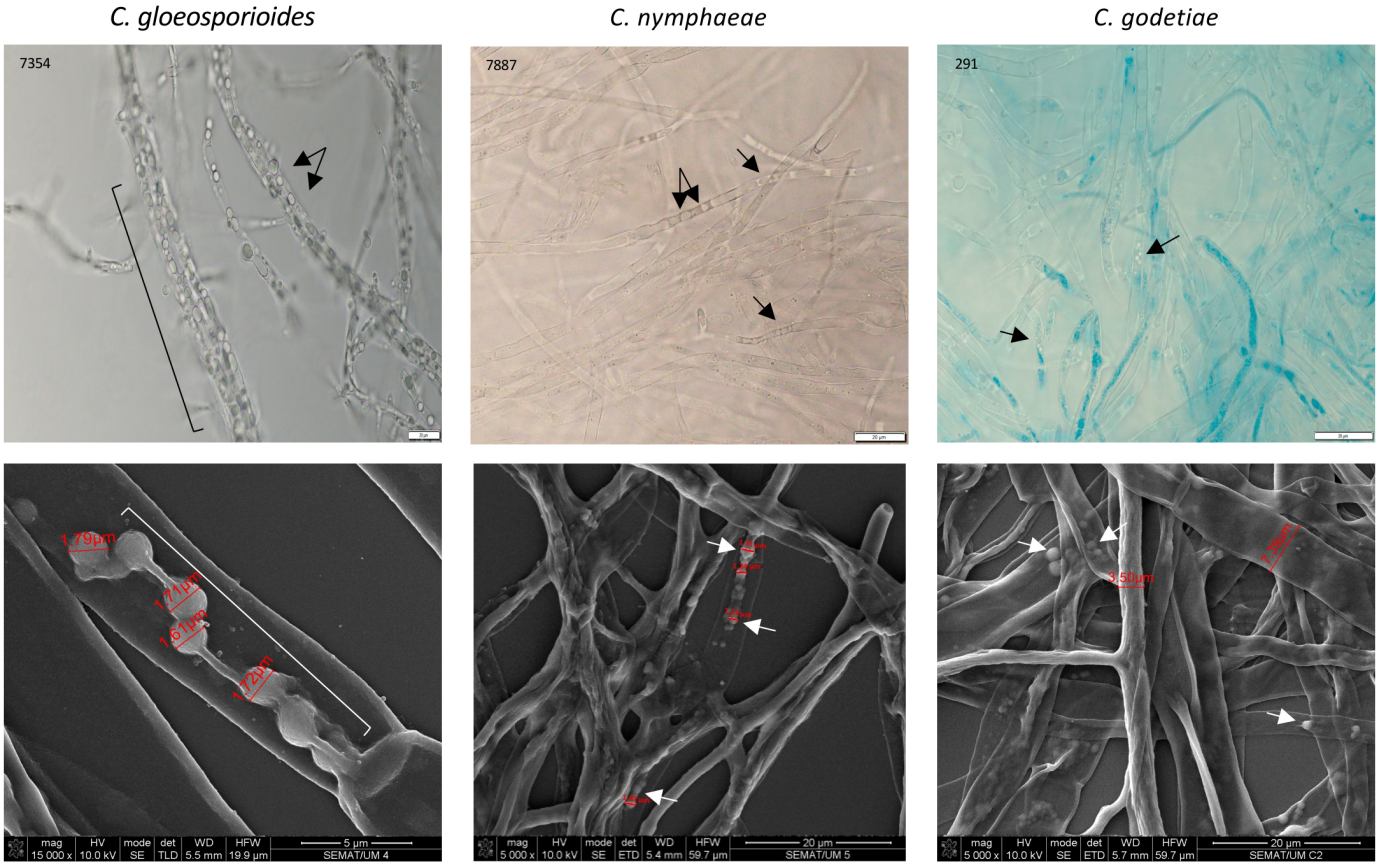
Light microscopy (upper panels) and SEM (lower panels) images of fungi cultivated without the presence of the yeasts. Arrows and brackets indicate spheroid structures inside the hyphae, much smaller than yeast cells.

*W. anomalus* #7, while antagonizing *M. perniciosa*, showed a *veil* covering the hyphae and yeast cells which was speculated to be precisely that (Ferraz et al., 2021). Additionally, Zepeda-Giraud et al. (2016) showed *W. anomalus* does produce biofilm although this ability was tested *in vitro* using monocultures and not in the presence of fungi. Presently, the secretion of a mucilage was very visible in *W. anomalus* co-cultured with any of the three *Colletotrichum* species (**Figure 6**, details in **Figure 7** upper panels). The ECM clearly forms viscous extensions connecting yeasts to one another, and yeasts with hyphae, forming a kind of net that is very similar to the yeast biofilms and colonies’ ECM previously reported (Kuthan et al., 2003). Another structure that was visible by SEM analysis of yeast-fungus co-cultures was that of yeast fimbriae (**Figure 6**, details in **Figure 7**). This has very seldom been described and there is no information on their structure, constitution, or the pathways and genes involved in their making. Previously, identical structures were described for *W. anomalus* #7 when facing *M. perniciosa* (Ferraz et al., 2021) or for *S. cerevisiae* cells within colonies (Varon and Choder, 2000). SEM observations further showed a slimy ECM/extracellular polymeric substance (EPS) covering and bridging yeast cells (**Figure 7**) that suggests that the yeast predation process has features in common with biofilm formation. Biofilms are associated with phytopathogenic fungi virulence, facilitating their colonization and development (Villa et al., 2017; Motaung et al., 2020). Furthermore, the prevention of these infections by endogenous or exogenous BCAs has been associated with the antagonizing organism’s ability to form biofilms (Liu et al., 2013; Verma et al., 2022). The biofilm coating most possibly generates a physical barrier that impedes the fungi from attaching and acceding the plant tissue. Additionally, the observation of samples from monocultures using SEM, as illustrated in **Figure 8**, revealed numerous small spheroid structures inside the hyphae displaying very heterogenous sizes (5.7 ± 4.9 µm^3^ (n=35)). The same structures, when observed in yeast/fungus co-cultures, could not be mistaken for yeasts inside the hyphae since they were too small and overlapped to be measured. They could therefore correspond to the micellization of hyphal contents preceding hyphal death. However, the yeasts attached to the hyphae measured in SEM micrographs were considerably smaller than when measured in light microscopy (6.27 ± 4.11 µm^3^ (n=40)), which may be attributed to the processes of sample fixation and dehydration (Frankl et al., 2015).

The possibility that *W. anomalus* kills and feeds on the fungal cells like a true predator has been proposed before for *M. perniciosa* antagonism (Ferraz et al., 2021). Other *W. anomalus* strains were previously reported to antagonize and kill *C. gloeosporioides* (Lima et al., 2013; Zepeda-Giraud et al., 2016) and the fungus *Botryodiploidia theobromae* (Hashem and Alamri, 2009). These authors showed that the yeast cells accumulated around the hyphae, adhering to their surface, pulling it inwards, and fusing with the hyphae cell wall (Hashem and Alamri, 2009; Lima et al., 2013; Ferraz et al., 2021). Additionally, some authors interpreted their SEM images as yeasts in the process of penetrating the hyphae (Hashem and Alamri, 2009; Lima et al., 2013), although this technique does not give real evidence of that occurrence. Also in the same direction, *C. gloeosporioides* was reported to be attacked in a similar fashion by *Debaryomyces nepalensis* (Zhou et al., 2018). In the present study, it was shown that *W. anomalus* #7 and *W. anomalus* #18 not only present these features but can also be found packing inside the hyphae, which confirms their predatory behavior towards *Colletotrichum*. In conclusion, *W. anomalus* presented an identical behavior while facing several phylogenetically distant fungi: *C. gloeosporioides* (this work; Lima et al., 2013; Zepeda-Giraud et al., 2016); *C. godetiae; C. nymphaeae* (this work); *B. theobromae* (Hashem and Alamri, 2009); and *M. perniciosa* (Ferraz et al., 2021). It antagonizes them essentially by close contact and, as shown in this work, adopts a predator-like behavior, to which the secretion of harmful compounds or enzymes or other antagonism mechanisms so often described (Passoth et al., 2006; Walker, 2011) may act only as ancillary mechanisms.

## 4 Final discussion

While some authors stress the influence of the yeast strain and cultivation conditions on the production of VOCs, siderophores, lytic enzymes, killer toxins, or other compounds (*e.g.*, Hoffmann et al., 2020), other authors question their actual individual or combined effect on inhibiting fungal growth (Contarino et al., 2019). *W. anomalus* LBCM1105 was previously shown to be able to strongly antagonize the cacao Witches Broom Disease causative agent, the fungus *M. perniciosa* (Ferraz et al., 2021). The present results showed that the same strain, when facing the OA-causing fungi, did not depend on those strategies to act as a BCA. Rather, it operated as a true microbial predator, possibly as a necrotrophic mycoparasite. This is evidenced by the adherence of the yeast cells to the hyphae, followed by the emptying of the hyphae. For this purpose, the yeast produced a mucous extracellular matrix-type substance that was found covering both the yeast and hyphal cells. In spite of *W. anomalus* forming biofilms, and in spite of the fact that the formation of a biofilm is considered by some authors a pre-requisite for considering a microorganism a BCA, a true biofilm was not actually formed. Moreover, penetration of the hyphae, or even only its emptying, should benefit from the degradation of the fungal cell wall by hydrolytic enzymes, which are produced by the yeast in very low amounts regardless of the presence of the fungi. Importantly, yeast-invaded hyphae were observed in co-cultures with *C. godetieae.* Co-cultures of *C. gloeosporioides* and *C. nymphaeae* equally displayed large amounts of empty hyphae, suggesting the yeasts might be able feed on the hyphal contents, but no yeasts were found inside them. This is was corroborated by the fact that yeasts stayed viable and reproductive after being co-cultured for long periods of time, which was not expected from regular monocultures. All considered, *W. anomalus*, particularly the strain LBCM 1105 (#7), actively and strongly preyed upon the three OA-causing fungi. *W. anomalus* is not GRAS (Generally Regarded as Safe), but it is a QPS (Qualified Presumption of Safety) microorganism (Sundh and Melin, 2010). This classification is restricted to a few uses in the food industry, including the post-harvest preservation of fruits and vegetables, therefore not including the release of the yeast into the environment. Yet, recently, *W. anomalus* was subjected to safety tests bearing in mind its possible utilization as an anti-plasmodium organism in the management of malaria and other human vector-borne diseases (Cappelli et al., 2021). Expectations are therefore generated that it could be used for phytosanitary purposes as well.

## Data Availability

The original contributions presented in the study are included in the article/**Supplementary Material**. Further inquiries can be directed to the corresponding author.

## Supplementary material

Supplementary Figure 1 Growth rates of *C. gloeosporioides*, *C. godetiae* and *C. nymphaeae* in MEA (light grey) and PDA (dark grey), at several initial media pH, at 25°C **(A, C, D)**. *C. gloeosporioides* was further assayed at 30°C **(B)**. The results are the median and standard deviation of ≥ 9 independent assays. The results obtained in MEA differ significantly from those obtained in PDA (*p*-*value <*0.05). Letters indicate statistically significant differences across different pH values in each media/temperature.

Supplementary Figure 2 Results of enzymatic activity of β-glucanase and chitinase in the cell-free extracts of *W. anomalus* #7 and fungi co-cultures supernatants. No significant differences were found between all the results of β-glucanase activity (F(5.48)= 0,457, *p-value* 0.806). The results of chitinase activity from the yeast-alone control were significantly different from those from *W. anomalus/C. godetiae* co-cultures (F(5.48)= 3.814, *p-value* 0.0055). The remaining results were not significantly different from each other.
