# Supplementary figures and images for: The yeast Wickerhamomyces anomalus acts as a predator of the olive anthracnose-causing fungi, Colletotrichum nymphaeae, C. godetiae, and C. gloeosporioides
Source: Front Fungal Biol. 2024 Sep 17;5:1463860. doi: 10.3389/ffunb.2024.1463860 (PMC11443700; doi:10.3389/ffunb.2024.1463860)

Fig. S1

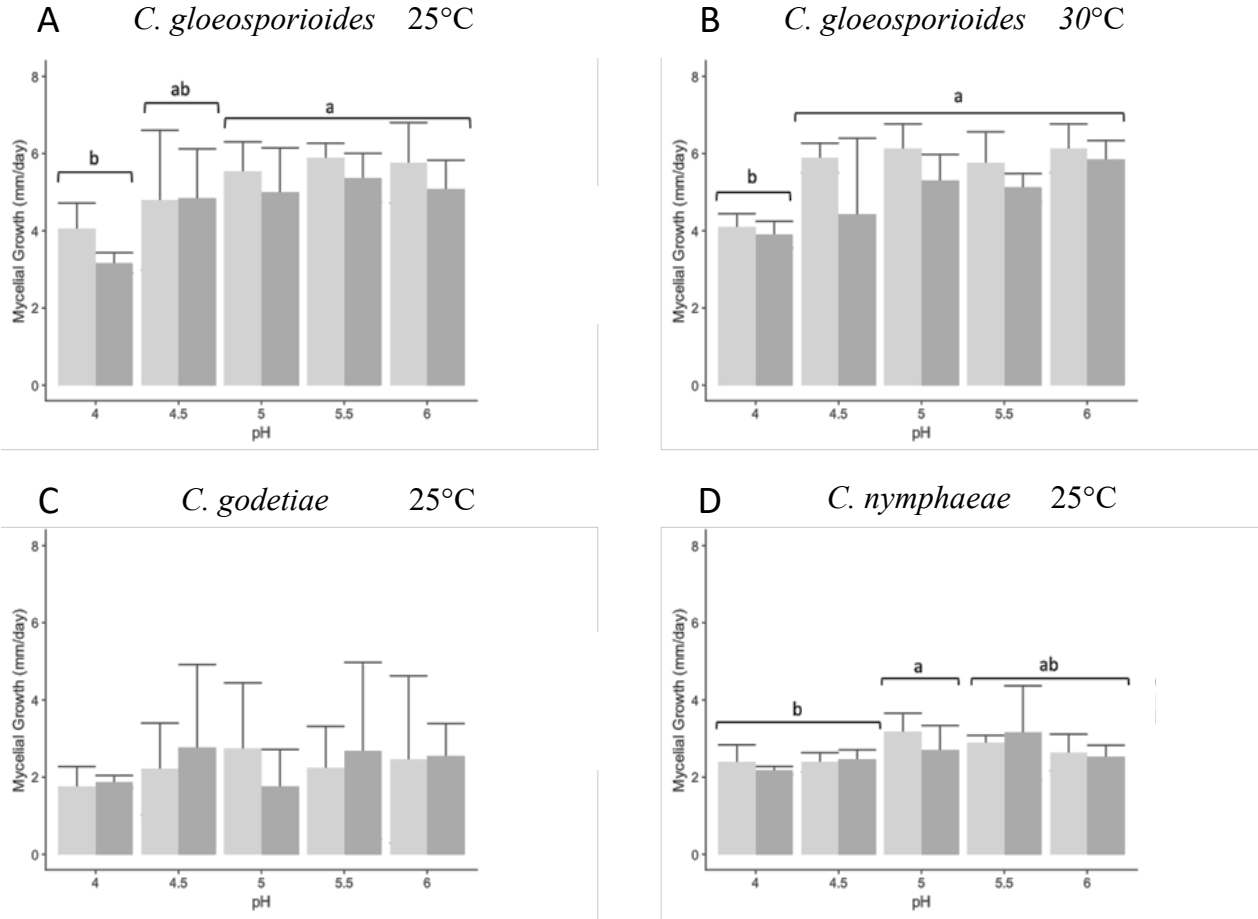

Supplement: Supplementary Figure 1 — Growth rates of C. gloeosporioides, C. godetiae and C. nymphaeae in MEA (light grey) and PDA (dark grey), at several initial media pH, at 25°C (A, C, D). C. gloeosporioides was further assayed at 30°C (B). The results are the median and standard deviation of ≥ 9 independent assays. The results obtained in MEA differ significantly from those obtained in PDA (p-value <0.05). Letters indicate statistically significant differences across different pH values in each media/temperature. [file Image1.pdf]

Fig. S2

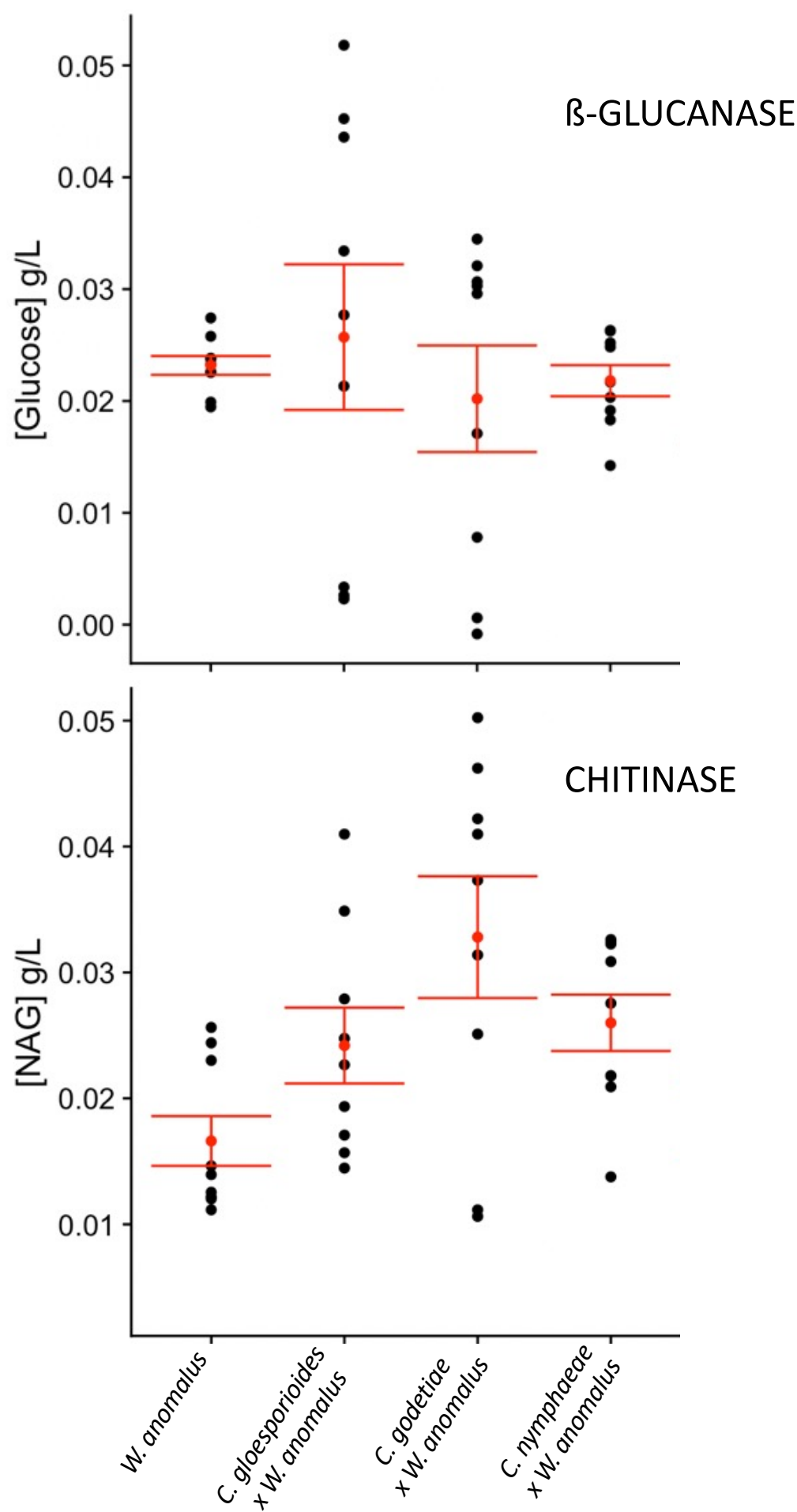

Supplement: Supplementary Figure 2 — Results of enzymatic activity of β-glucanase and chitinase in the cell-free extracts of W. anomalus #7 and fungi co-cultures supernatants. No significant differences were found between all the results of β-glucanase activity (F(5.48)= 0,457, p-value 0.806). The results of chitinase activity from the yeast-alone control were significantly different from those from W. anomalus/C. godetiae co-cultures (F(5.48)= 3.814, p-value 0.0055). The remaining results were not significantly different from each other. [file Image2.pdf]
