# Supplementary material for: The yeast Wickerhamomyces anomalus acts as a predator of the olive anthracnose-causing fungi, Colletotrichum nymphaeae, C. godetiae, and C. gloeosporioides
Source: Front Fungal Biol. 2024 Sep 17;5:1463860. doi: 10.3389/ffunb.2024.1463860 (PMC11443700; doi:10.3389/ffunb.2024.1463860)
Supplement: Supplementary file 3 [file Table1.pdf]

Table S1. Degree of antagonism in liquid medium between the yeast strains in Table 1 and phytopathogenic *Colletotrichum* species. Results (n = 3 to 6) are expressed using an empirical scale previously developed (Ferraz et al., 2021), of 0-1-2 (from full mycelium growth to no growth), after 8 days incubation at 25°C.

	**Yeast industrial strains**								
	**#1**	**#2**	**#3**	**#4**	**#5**	**#6**	**#7**	**#8**	**#9**
C. gloeosporioides s.s.	2	0	0	0	2	2	2	2	2
C. godetiae	2	1	0	0	2	2	2	2	2
C. nymphaeae	2	0	0	0	2	2	2	2	2

	**Yeast strains collected from infected orchards**											
	**#10**	**#11**	**#12**	**#13**	**#14**	**#15**	**#16**	**#17**	**#18**	**#19**	**#20**	**#21**
C. gloeosporioides s.s.	2	1	2	2	2	2	0	1	2	1	2	0
C. godetiae	2	2	2	2	2	2	0	0	2	1	2	1
C. nymphaeae	2	1	2	2	2	2	0	1	2	1	2	0
